# Multivariate Optimization of Pb^2+^ Adsorption onto Ethiopian Low-Cost Odaracha Soil Using Response Surface Methodology

**DOI:** 10.3390/molecules26216477

**Published:** 2021-10-27

**Authors:** Yohanis Birhanu, Seyoum Leta

**Affiliations:** 1 Department of Chemistry, College of Natural and Computational Science, Jigjiga University, Jigjiga P.O. Box 1020, Ethiopia; 2 Center of Environmental Science, Addis Ababa University, Addis Ababa P.O. Box 1176, Ethiopia; leta.seyoum@aau.edu.et

**Keywords:** odaracha, wastewater, lead, adsorption, response surface method

## Abstract

Lead pollution is a severe health concern for humankind. Utilizing water contaminated with lead can cause musculoskeletal, renal, neurological, and fertility impairments. Therefore, to remove lead ions, proficient, and cost-effective methods are imperative. In this study, the Odaracha soil which is traditionally used by the local community of the Saketa District was used as a novel low-cost technology to adsorb lead ions. Odaracha adsorbent was characterized by scanning electron microscopy and Fourier transform infrared spectroscopy. The adsorption process followed the batch adsorption experiment. The response surface method was implemented to derive the operating variables’ binary interaction effect and optimize the process. According to the study’s experimental result, at optimum experimental conditions Odaracha adsorbent removes 98.17% of lead ions. Based on the result of the central composite design model, the Pb^2+^ ion removal efficiency of Odaracha was 97.193%, indicating an insignificant dissimilarity of the actual and predicted results. The coefficient of determination (R^2^) for Pb^2+^ was 0.9454. According to the factors’ influence indicated in the results of the central composite design model, all individual factors and the interaction effect between contact time and pH has a significant positive effect on lead adsorption. However, other interaction effects (contact time with dose and pH with dose) did not significantly influence the removal efficiency of lead ions. The adsorption kinetics were perfectly fitted with a pseudo-second-order model, and the adsorption isotherm was well fitted with the Freundlich isotherm model. In general, this study suggested that Odaracha adsorbent can be considered a potential adsorbent to remove Pb^2+^ ions and it is conceivable to raise its effectiveness by extracting its constituents at the industrial level.

## 1. Introduction

Lead pollution is a serious environmental concern, and lead is a toxic inorganic pollutant in surface and groundwater, even at trace levels [1]. However, battery, pigment, printing, fuel, and photographic manufacturing activities demand lead. These sources are the principal causes of human exposure to lead. Children are greatly affected by lead poisoning because 99% of the lead that enters the adult, whereas only 33% that enters the child’s body, are excreted in about two weeks [2]. Because of its nonbiodegradability nature, lead can be accumulated in different parts of the human body. Lead contamination accumulation is mostly in human bones and teeth, which causes bone weakness in joints and fingers [3]. Contamination of lead can also cause musculoskeletal, visual, renal, neurological, fertility impairment, and hypertension problems [4,5].

In removing lead and other heavy metals, coagulation, electrolysis, precipitation, reverse osmosis, adsorption, and ion exchange methods are used [6,7,8,9,10,11]. Each removal technology has its limitations and advantage in terms of operational cost, production of secondary contaminants, the volume of sludge produced, working speed, and applicability at the large scale or industrial level [7]. However, clay minerals and other natural adsorbents are preferable for heavy metal adsorption because of their low cost, abundance, net negative charge, high surface area, best performance, and potential for ion exchange [12,13,14,15,16]. The negative charges on the surface of clay particles play an important role in binding positively charged atoms or molecules but allow these to exchange with other positively charged particles. Therefore, as the net negative charge of the adsorbent rises, its cation exchange capacity also increases.

Mostly, the solubility of soil particles causes the turbidity problems of water bodies. However, on the contrary, the physicochemical property of the Odaracha soil helps to remove the suspended particles from water. As a result, Odaracha soil is considered a special, prominent natural material with the local community of the Saketa district, Ethiopia. In some rural parts of the West Harerghe zone of Oromia Regional State, people utilize this material to clarify water with a high turbidity level and commercialize it in the market, especially in rural vicinities where scarcity of drinking water is common [17]. Even though the studies have been conducted on chromium and turbidity removal, there is no scientific investigations about the potential of Odaracha soil in lead removal. Therefore, this study’s primary objective was to explore the effectiveness of Odaracha soil in removing Pb^2+^ from synthetic wastewater by considering the effect of contact time, pH, adsorbent dose, and initial concentration of lead. Adsorption isotherm and kinetic studies are also involved to describe the movement of a substance from aqueous media to a solid phase and to understand the rate of the adsorption process.

## 2. Materials and Methods

### 2.1. Adsorbent Preparation and Characterization Techniques

Odaracha soil was obtained from the Saketa district West Harerghe Zone of Oromia Regional State, Ethiopia (N: 08°44′41.3″ E: 040°45′15.1″, Altitude 1470). The soil sample was used without further modification. The Odaracha sample was crushed and sieved using a standard laboratory sieve of 120 µm mesh, and to enhance the active sites of the adsorbent it was dehydrated at 120 °C for approximately 6 hrs. The Odaracha adsorbent was subjected to scanning electron microscopy analysis using an INSPECT F50 field emission scanning electron microscope to observe surface morphology changes before and after adsorption [18]. The functional groups involved in the adsorption of lead ions were identified by Fourier transform infrared (FTIR) spectroscopy (Model 65 spectrometer, USA) [19]. The FTIR spectra ranged from 400 cm^−1^ to 4000 cm^−1^. Soil texture was determined by the hydrometer method.

To determine the pH of point zero charge (pHpzc), 50 mL of 0.1 M of NaNO_3_ solution was transferred into the laboratory flasks. A pH adjustment from 1 to 10 pH levels was made by the addition of 0.1 M of NaOH or 0.1 M of HNO_3_. Then, in each flask, 1 g of Odaracha adsorbent was added. Finally, the suspension was equilibrated, and the final pH value of the solution was recorded. A graph of pH_o_—pH_f_ (*y*-axis) against pH_o_ (*x*-axis) was plotted. In this graph, the point where the graph intersects the *x*-axis is taken as the pH of the point of zero charge.

### 2.2. Batch Adsorption Experiments

All the required solutions were prepared with analytical-grade reagents. A 1000 ppm standard stock solution of Pb^2+^ was prepared by dissolving 1.615 g of 99% Pb(NO_3_)_2_ in double-distilled water in a 1 L volumetric flask. Synthetic samples of different lead concentrations were also prepared from this stock solution by appropriate dilutions. Lead stock solutions (30 mg/L) were prepared by diluting 30 mL of 1000 ppm lead standard solution in 1000 mL of distilled water using a volumetric flask. Correspondingly, metal ion concentrations having 50, 70, 90, 110, 130, and 150 mg/L were prepared. The aqueous solution’s pH was adjusted to the desired value by adding a 0.1 N HNO_3_ or a 0.1N NaOH solution. The adsorption experiments were conducted at room temperature, with 150 rpm agitation speeds in Erlenmeyer flasks. The adsorption capacity of Odaracha soil to adsorb Pb^2+^ ions from the wastewater was studied under various experimental conditions of pH, contact time, adsorbent dose, and initial lead concentration. The concentration of Pb^2+^ ions after adsorption was determined using FAAS (Atomic Absorption Spectrophotometer, Model 210 VGP) for the solution’s remaining lead ions. All the batch adsorption experiments were carried out in triplicate. The adsorption percentage of lead was computed with Equation (1) and the amount of adsorbed lead ion per unit mass of sorbent (*q_e_*) was calculated using Equation (2).

(1) $$\%\;removal=\frac{\left(C_{o}-C_{e}\right)}{C_{o}}\times 100$$

where *C_o_* = initial concentration of lead ion (mg/L) and *C_e_* = final equilibrium adsorbate concentration (mg/L).

(2) $$q_{e}=\frac{\left(C_{o}-C_{e}\right)V}{w}$$

*C_o_* is the Pb ions’ initial concentrations in mg L^−1^, and *C_e_* represents equilibrium liquid-phase concentrations of the Pb ions in mg L^−1^. *V* is the volume of the solution in L, *w* is the amount of Odaracha soil measured in g, and *q_e_* is lead removal efficiency of the adsorbent in mg/g.

### 2.3. Response Surface Method (RSM)

In this study, response surface methodology (RSM) was used to develop a mathematical model to study the impacts of the response’s main operative parameters. The study observed the effects of the main operative parameters on the adsorption of lead using Odaracha adsorbent. In this regard, the process was modeled and optimized by considering three parameters: contact time, pH, and adsorbent dose, each measured at three levels using a central composite design (CCD). CCD is known as one of the primary design techniques in RSM. This mathematical model was used to build a second-order model (quadratic model), and is typical for process optimization. Design-Expert software version 12.0.7.0 (Stat-Ease, Suite 6400, Minneapolis, MN 55413, USA) was used to construct the mathematical model. A quadratic polynomial model Equation (3) was used to correlate the response and independent variables (operational factors).

(3) $$Y=\beta_{o}+\sum_{i=1}^{k}\beta_{i}X_{i}+\sum_{i=1}^{k}\beta_{ii}X^{2}{}_{i}+\sum_{i=1}^{k-1}\sum_{j=i+1}^{k}\beta_{ij}X_{i}X_{j}+\varepsilon$$

where *Y* is the predicted response model (Pb^2+^ removal percentage), $\beta_{o}$ is the constant coefficient, $\beta_{i}$ is the coefficient of the linear term, $\beta_{ii}$ is the interactive coefficient, $\beta_{ij}$ is the coefficient of the quadratic term, k is the number of experimental factors and $X_{i}$ and $X_{j}$ are the coded values of the experimental factors.

Analysis of variance checked the model accuracy and the input parameters’ effects on the response variable through a statistical evaluation of the P-value and F-value of the regression coefficient at a 95% confidence interval. The coefficient of determination (R^2^), the adjusted coefficient of determination (R^2^_adj_), adequate precision (AP), and the coefficient variation (CV) were used to assess the fitness quality of the developed model. Three-dimensional response surface plots displayed the interaction between independent factors and their respective effect on the response variable.

## 3. Result and Discussion

### 3.1. Characterization of Odaracha Adsorbent

#### 3.1.1. Scanning Electron Microscopic (SEM) Studies

**Figure 1:**
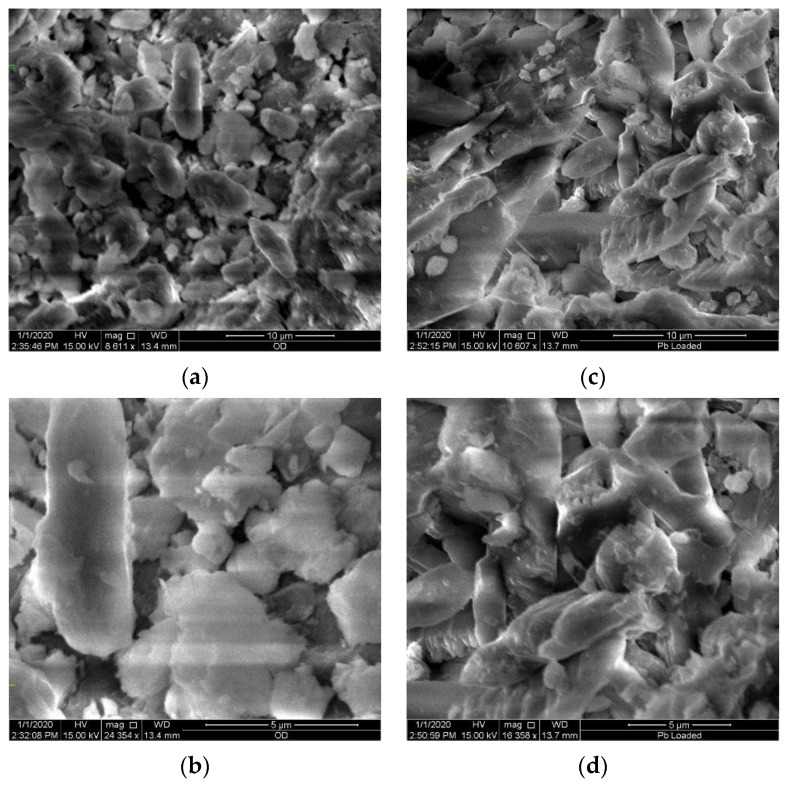
SEM images of Odaracha adsorbent before (**a**,**b**) and after adsorption (**c**,**d**) of lead.

As indicated in the SEM microgram in Figure 1, the surface of Odaracha soil appeared rough and porous before lead ion adsorption; this is the reason for lead ion adsorption. As illustrated in Figure 1c,d, following the adsorption study, the pores and the soil surface caves became filled and smooth, which indicated the adsorption of lead ions on the surface of Odaracha soil.

#### 3.1.2. FT-IR Studies

**Figure 2:**
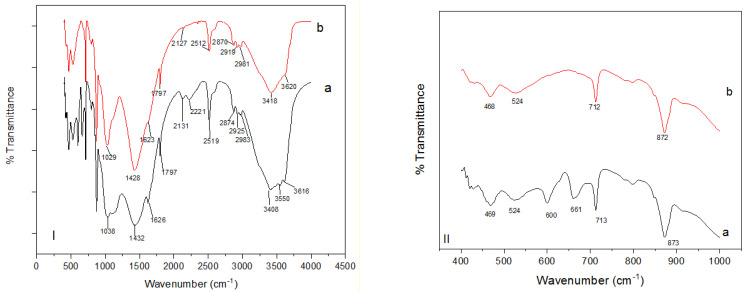
FTIR spectrum (400–4000 cm^−1^ (**I**) and 400–1000 cm^−1^ (**II**)) of raw (a) and lead-loaded (b) Odaracha powder.

Figure 2 depicts the FT-IR spectra of Odaracha soil. FTIR bands before and after lead adsorption were produced to show the functional groups that participated in lead ion adsorption. When a ligand combines with a metal, the disturbance of ligand material’s energy occurs, causing changes in the FTIR spectra absorption peaks [17]. These peaks are usually reduced to lower or increases to upper frequencies. Consequently, there was a small shift of vibration ring at 469, 873, 1038, 1432, 1797, 2519, 3408, and 3616 cm^−1^ in the Odaracha adsorbent to 467, 871, 1026, 1427, 1795, 2513, 3421, and 3620 cm^−1^ for the Pb^2+^-loaded adsorbent. The peaks at 600, 661, 1149, 1626, 2125, and 2215 cm^−1^ disappeared. The removal of intensities and alteration of band values signify the functional groups’ involvement in the adsorption process. The small, shifted picks around 3400 and 3600 cm^−1^ after adsorption of lead ions signifies the involvements of -OH stretching vibrations. The small, shifted and absent picks at around 1000, 1400, and 2500 cm^−1^ indicate the involvement of silica. However, the spectra below 1000 cm^−1^ are more related to the mineral features of the adsorbent [20].

#### 3.1.3. Physical and Chemical Property of Odaracha Soil

Different oxides such as silica, aluminum oxide, ferric oxides, and titanium oxide are extensively used for heavy metal removal [21,22,23,24]. Their morphology makes them promising oxides for heavy metal adsorption, and plays a significant role in their adsorption capacity [25,26]. According to the result indicated by [27], the proportions of SiO_2_, Al_2_O_3_, Fe_2_O_3_, MgO, MnO, and TiO_2_ were 30.94, 8.71, 5.18, 1.44, 0.26 and 0.3%, respectively.

According to the soil physical property result indicated in Table 1, the texture type of Odaracha soil is categorized as clay.

#### 3.1.4. Determination of the pH of the Point of Zero Charge (pHpzc)

**Figure 3:**
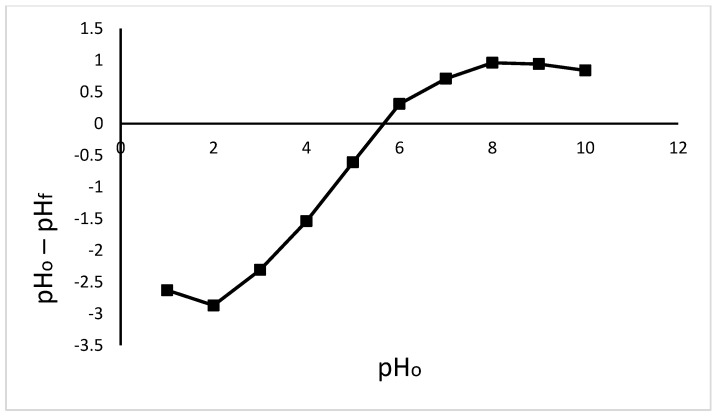
Point zero charge (pH_pzc_) of Odaracha adsorbent.

According to the results shown in Figure 3, the pHpzc of the Odaracha adsorbent was 5.6. In this regard, when the solution pH is less than 5.6, the surface of the Odaracha adsorbent becomes positively charged, having a greater affinity for anions. When it is above pHpzc, the surface of the adsorbent becomes negatively charged, which creates a situation conducive for the adsorption of cationic species [28,29]. In this study, the maximum adsorption of lead was recorded at a pH of 6. At pH 6, the surface of Odaracha is anticipated to be negative. Therefore, the point zero charge value supports the experimental results of lead adsorption making the adsorbent amenable for lead ion adsorption by means of electrostatic attraction.

### 3.2. Effect of Various Factors on the Adsorption of Pb^2+^

The Pb removal efficiencies of Odaracha adsorbent were investigated based on the following equations. The percentage of lead (II) removal was obtained using Equation (1) while the adsorption capacity *q_e_* was calculated with Equation (2).

#### 3.2.1. Effect of Contact Time

Contact time is an essential factor in the adsorption of Pb (II) [30]. A study of the effect of contact time can show the diffusion rate of adsorbate in the solid-liquid adsorption system and help to determine the optimum adsorption period of the adsorbent [31]. As illustrated in Table 2, the effect of contact time varied from 60 to 240 min. Adsorption efficiency of Odaracha adsorbent was carried out under constant experimental conditions including pH (pH = 6), adsorbent dose (15 g/L), agitation speed (150 rpm) and initial concentration of adsorbate (70 mg/L).

According to the results in Table 2, the adsorption percentage of Pb (II) increased with increased contact up to 180 min and remained constant at 240 min. A similar trend was perceived with Pb^2+^ uptake in mg/g, which increased with contact time up to 180 min and remained unchanged significantly after 180 min. The majority (91.565%) of lead adsorption occurred within the first 60 min, and was attributed to the presence of lots of active sites on the adsorbent surface at the beginning [32]. The optimum contact time for maximum adsorption of Pb (II) was 180 min. Subsequently, there was an insignificant decreasing trend. As shown in Table 2, the highest adsorption percentage and lead uptake in mg/g at 180 min was 97.194% and 4.536 mg/g, respectively.

#### 3.2.2. Effect of pH on Adsorption of Pb(II)

The pH of the solution is an essential controlling parameter in the adsorption of Pb^2+^. The adsorption capacity of Pb^2+^ was strongly affected by the variation of pH of the solution [31,33]. In this study, the effect of pH on Pb (II) adsorption efficiency was studied by varying pH levels from 3 to 6 with a one-unit interval. At pH values greater than 6, precipitation is dominant, or metal hydroxide formation becomes a significant mechanism in the metal removal system [34]. This condition is not desirable as the metal precipitation could lead to a misunderstanding of the adsorbent’s adsorption capacity. Because metal hydroxides are generally not stabilized forms of heavy metal, precipitation can sometimes decompose upon the effluent’s neutralization from the wastewater treatment plant. As a result, the solubility of the metals increases recontamination by metal ions.

In this study, the effect of pH on Pb (II) adsorption efficiency was undertaken with a 120 min contact time, 15 g/L of adsorbent dose, 70 mg/L of adsorbate concentration, and 150 rpm agitation speed. As observed in Table 3, the adsorption percentage of Pb (II) by Odaracha adsorbent increased while the pH of the solution increased from 3 to 6. This incremental trend of adsorption capacity of Pb^2+^ with growing solution pH was confirmed by previous studies [12]. Therefore, in this experimental setup, pH 6 was the optimum solution pH that exhibited the maximum adsorption percentage of Pb^2+^. As revealed in Table 3, at the optimum pH condition, the highest adsorption percentage and uptake in mg/g of Pb^2+^ by Odaracha adsorbent was 92.007% and 4.291 mg/g, respectively. The lowest adsorption percentage and uptake in mg/g were obtained at pH 3, which were 89.711%, and 4.187 mg/g, respectively, and may be due to the competitive influence of H^+^ ions. H^+^ ions compete with the metal ions for the active sites on the adsorbent, causing the reduction of adsorption of Pb (II) ions [35]. At pH 3, the concentration of H^+^ is high, which promotes the protonation of functional groups of the adsorbent. It makes the adsorbent more positively charged, leading to electrostatic repulsion of positively charged Pb^2+^ ions [36].

#### 3.2.3. Effect of Adsorbent Dose on Adsorption Efficiency of Pb(II)

Experiments with the use of different dosages of Odaracha adsorbent at 1, 5, 10 and 15 g/L were carried out to evaluate the effect of adsorbent dosage on adsorption of Pb^2+^ ions under the optimum experimental conditions of 180 min contact time, 150 rpm agitation speed, 70 mg/L initial concentration of adsorbate and solution pH of 6.

The impact of the adsorbent dose was determined by varying its weight from 1 to 15 g/L. As shown in Table 4, the adsorption percentage of Pb^2+^ ions was found to increase with the increase of Odaracha adsorbent per liter of aqueous solution. The adsorption percentage of Pb^2+^ ions was increased from 66.752% to 97.194%, with an increase in the adsorbent dose from 1 to 15 g/L. Because of the intensification of active sites of the adsorbent, greater availability of the exchangeable sites, and surface area, the adsorption percentage of lead ions increased with an increase of adsorbent dosage [37,38,39]. Therefore, 15 g/L of the adsorbent dose was considered the optimum dose under the experimental conditions indicated in Table 4. The uptake result in Table 4 shows the converse association between adsorbent dose and uptake of Pb^2+^ ions in mg/g. As the adsorbent dose increased from 1 to 15 g/L, the uptake of lead ion decreased. According to Table 4, the minimum uptake was noted at 15 g/L of adsorbent dose, which was 4.536 mg/g. The maximum uptake (46.726 mg/g) was recorded at pH 6 with 1 g/L of adsorbent dose. The decreasing trend of uptake of lead ions in mg/g while increasing adsorbent dose was also found by other researchers [40].

#### 3.2.4. Effect of Initial Concentration of Adsorbate on Adsorption of Pb(II)

Lead adsorption is significantly influenced by the initial concentration of Pb (II) ions in aqueous solutions. In this study, the initial concentration of lead, which varied from 30 to 150 mg/L, was used to explore the effect of the initial concentration of Pb^2+^ ions on the adsorption efficiency of Odaracha adsorbent under constant experimental conditions. The effects of the initial concentration of lead ions on the adsorption percentage and uptake of Pb^2+^ ions in mg/g are presented in Table 5.

The effect of initial Pb(II) ion concentration on the adsorption process was studied at seven different initial concentrations of lead ions ranging from 30 to 150 mg/L at the optimal adsorbent dose (15 g/L), contact time (180 min), and pH (pH 6). Table 5 shows that the adsorption percentage of lead ions decreased when the initial concentration of lead ion increased because the adsorbent materials have limited active sites, and at a specific concentration, their active sites become saturated [41]; the uptake of lead in mg/g showed the opposite trend. Accordingly, the adsorption percentage of Odaracha adsorbent decreased from 98.18% (for 30 mg/L) to 85.28% (for 150 mg/L) on the other hand, the uptake of Pb^2+^ increased from 1.96 mg/g (for 30 mg/L initial concentration of Pb^2+^) to 8.53 mg/g (for 150 mg/L of initial concentration of Pb^2+^). Previous studies showed similar results [42].

### 3.3. Response Surface Method (RSM) of Pb^2+^ Adsorption

To measure the relationship between the response variable (Pb^2+^ removal) and the independent variables such as contact time, pH, and adsorbent dose, response surface methodology was employed. A central composite design (CCD) model indicated a quadratic model represented the correlation between the response variable and all factors. On the other hand, a summary of fit statistics demonstrated that adjusted and predicted R^2^ were close to each other the difference being less than 0.2 [43]. The coefficient of determination (R^2^) approached 1, and the precision that measures the signal to noise ratio indicates a good signal since it was greater than 4 [44,45]. As shown in Equation (3), the predicted response was calculated in terms of a second-order polynomial equation with single, interaction, and quadratic terms representing the final quadratic model for the adsorption percentage of lead.

Y_Pb Removal_ = + 0.0111 + 0.0003 A + 0.0001 B + 0.0001 C + 0.000 AB + 0.0001 AC − 6.293E-07 BC − 0.0002 A^2^ + 0.0001 B^2^ − 0.0000 C^2^ (4)

**Figure 4:**
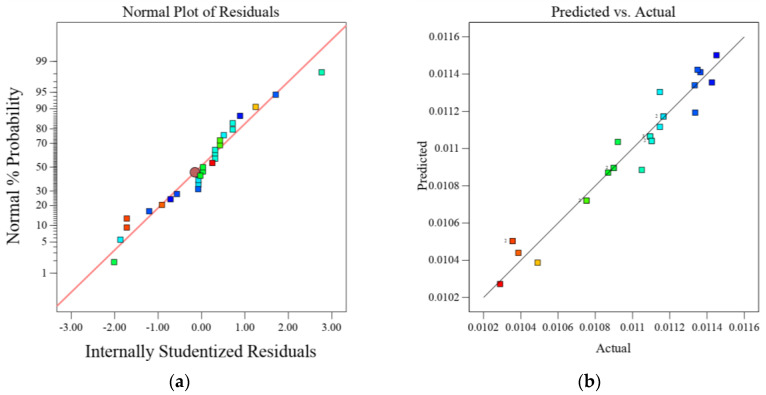
Residual plots: (**a**) normal probability, (**b**) predicted versus actual for removal of lead by Odaracha.

In the mathematical model, A, B, and C are independent singular factors, whereas AB, AC, and BC are interactional factors, and the quadratic terms include A^2^, B^2^, and C^2^. According to the evaluation results of the analysis of variance demonstrated in Table 6, the p-values for the individual parameters, interaction factors, and the quadratic terms such as A, B, C, AB, A^2^, and B^2^, were significant. However, the p-values of other interaction factors and quadratic terms such as AC, BC, and C^2^ were greater than 0.05. The RSM model can be considered reproducible when the coefficient of variance (CV) value is less than 10% [46]. In this regard, the value of the coefficient of variance of this study was 0.9143, which infers the model’s reproducibility. As demonstrated in Figure 4, the RSM model’s adequacy is also evaluated by residual diagnostic plots, which are the difference between the observed and predicted responses [47]. Accordingly, the experimental data were fitted in the RSM model to establish the relationship between the observed and predicted values. As illustrated in Figure 4a, all the data points are distributed near the straight line, indicating the quadratic model could be a useful model to predict the response. As indicated in Figure 4b, the predicted and actual value plots were close to each other, indorsing the suitability of the model.

**Figure 5:**
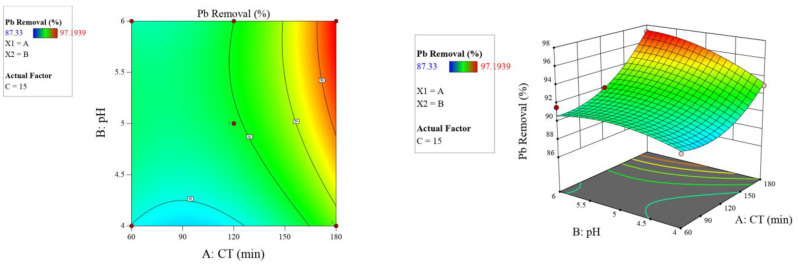
Two-dimensional contour and three-dimensional surface plots on the relation between contributed factors (contact time and pH) and Pb^2+^ removal by Odaracha adsorbent.

**Figure 6:**
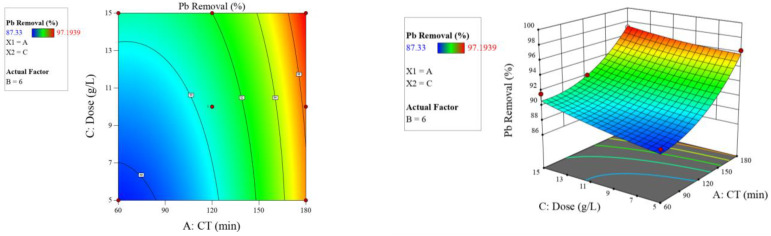
Two-dimensional contour and three-dimensional surface plots on the relation between contributed factors (contact time and dose) and Pb^2+^ removal by Odaracha adsorbent.

**Figure 7:**
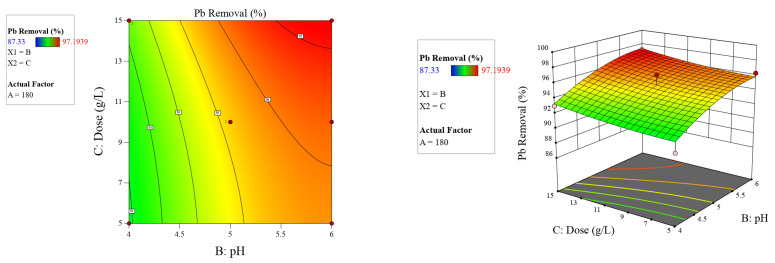
Two-dimensional contour and three-dimensional surface plots on the relation between contributed factors (pH and dose) and Pb^2+^ removal by Odaracha adsorbent.

As illustrated in Figure 5, Figure 6 and Figure 7, the 2D contour and 3D response surface plots demonstrate the independent variables’ interaction effects on the response. According to the factors’ influence indicated in Table 6, Figure 5, Figure 6 and Figure 7, and Equation (4), all individual factors and the interaction between contact time and pH had significant positive effects on lead adsorption.

### 3.4. Kinetic Study for Adsorption of Pb(II)

#### 3.4.1. Pseudo First Order Kinetic Model


(5) $$\log(q_{e}-q_{t})=logq_{e}-\frac{k_{1}t}{2.303}$$

In Equation (5) $q_{e}$ represents the amount of Pb^2+^ ions adsorbed per unit weight of the adsorbents at equilibrium, $q_{t}$ $\left({\mathrm{mg}\;g}^{-1}\right)$ represents the amount of Pb^2+^ ions adsorbed per unit weight of the adsorbents at time t (min), and the rate constant of the pseudo-first-order kinetic model is represented by k1.

**Figure 8:**
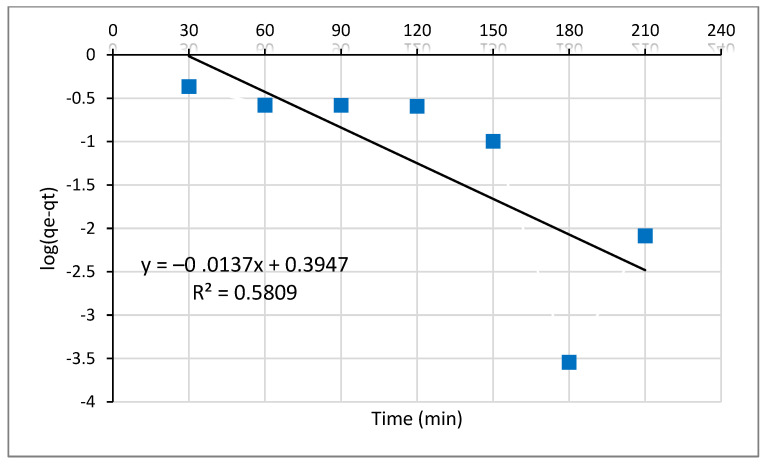
Pseudo-first-order kinetics plots for the adsorption of Pb^2+^ ions onto the Odaracha adsorbent.

The plot in Figure 8 shows that lead (II) adsorption with different contact times consisted of two-stages; a rapid initial stage where the adsorption was fast and a slower second stage where the adsorption equilibrium was achieved. If the plot of log $\left(q_{e}-q_{t\;}\right)$ against time (t) shows a linear relationship, the pseudo-first-order is appropriate, the rate constant (k1), $q_{e}$ (cal) and the correlation coefficient (R^2^) being determined from the straight-line plot of the log $\left(q_{e}-q_{t\;}\right)$ versus t. The values of k1 and $q_{e}$ (cal) in mg/g of Pb^2+^ predicted from the plot shown in Figure 8 are 3.155 × $10^{-2}$ and 2.48 mg/g, respectively.

The plot of log $\left(q_{e}-q_{t}\right)$ against time (t) presented in Figure 8 doesn’t display a linear relationship that communicates the pseudo-first-order model’s unsuitability. Furthermore, the experimental result of adsorption equilibrium ($q_{e}$ (exp)) which is 4.536 ${\mathrm{mg}\;g}^{-1}$ was not closer to the calculated result of adsorption equilibrium $q_{e}$ (cal), which is 2.48 ${\mathrm{mg}\;g}^{-1}$. Therefore the pseudo-first model is not suitable to explain the adsorption kinetics of Pb (II) ions on Odaracha adsorbent [48,49].

#### 3.4.2. Pseudo Second Order Kinetic Model

**Figure 9:**
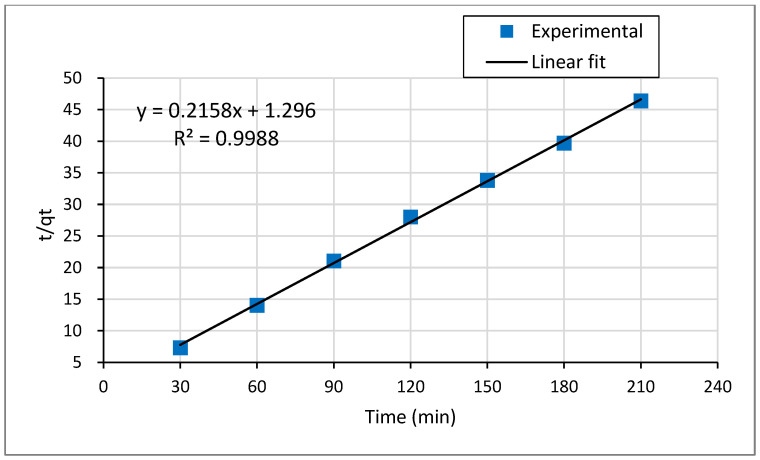
Pseudo-second-order kinetics plots for the adsorption of Pb^2+^ ions onto Odaracha adsorbent.

The pseudo-second-order model is expressed by Equation (6):

(6) $$\frac{t}{q_{t}}=\frac{1}{K_{2}q_{e}{}^{2}}\;+\frac{t}{q_{e}}$$

where $q_{e}$ and $q_{t}$ $\left({\mathrm{mg}\;g}^{-1}\right)$ represents the amount of Pb^2+^ ions adsorbed per unit weight of the adsorbent at equilibrium and time t (min). *K_2_* represents the pseudo-second-order rate constant. The rate constant (*k_2_*) and calculated equilibrium adsorption capacity ($q_{e}$ (cal)) can be measured from the slope and intercept of the plot $t/q_{t}$ versus t, which is shown in Figure 9. To apply the pseudo-second-order kinetic model, the plot $t/q_{t}$ against t must be a straight line. The plot of $t/q_{t}$ against t for pseudo-second-order shown in Figure 9 yields an excellent straight line with the highest correlation coefficient result approaching 1 (R^2^ = 0.9988). Moreover, as presented in Table 7, the experimental adsorption equilibrium value ($q_{e}$ (exp.) = 4.536 ${\mathrm{mg}\;g}^{-1}$) was well matched with the calculated adsorption equilibrium value ($q_{e}$ (cal.) = 4.6339 ${\mathrm{mg}\;g}^{-1}$). Thus, the adsorption kinetics of lead ions is accurately supported by the pseudo-second-order model. Therefore, this study endorses that the rate-limiting feature in the adsorption of lead(II) by Odaracha adsorbent is chemisorption involving the exchange of Pb^2+^ ions with functional groups in the adsorbent [32,50].

### 3.5. Isotherm Model for Adsorption of Pb(II)

In a solid-liquid adsorption system, the adsorption isotherm model defines the adsorption behavior [51]. Therefore, the distribution of the Pb^2+^ ion onto the adsorbent surface was determined by the Langmuir and Freundlich adsorption isotherm models.

#### 3.5.1. Langmuir Adsorption Isotherm Model for Pb^2+^

The Langmuir model is the well-known monolayer adsorption isotherm model that relates the adsorbate’s equilibrium concentration with that of the adsorbent’s adsorption capacity.

(7) $$\frac{1}{q_{e}}=\frac{1}{q_{m}}+\frac{1}{q_{m}K_{L}}\;.\frac{1}{C_{e}}\ldots$$

where *q_e_* represents the amount of adsorbed Pb^2+^ in mg/g of the adsorbent, and *C_e_* is the concentration of Pb^2+^ at equilibrium in mg/L. *K_L_* and *q_m_* symbolize the Langmuir adsorption constant and the maximum amount of adsorbate that can be adsorbed on Odaracha adsorbent [52].

**Figure 10:**
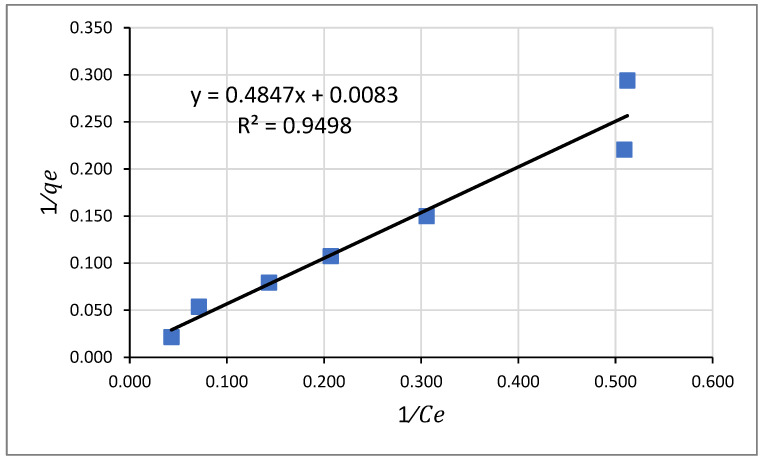
Langmuir Adsorption Isotherm Model for Lead (II).

As revealed in Figure 10, the equilibrium adsorption data for lead removal using Odaracha adsorbent was fitted into the linear form of Langmuir’s Equation (7) to determine the distribution of Pb^2+^ ions on the surface of Odaracha soil. Additional analysis of Langmuir isotherm was made using a dimensionless parameter (*R_L_*) shown in Equation (8). Equation (8) was used to predict whether the adsorption process is favorable (0 < *R_L_* < 1) or unfavorable (*R_L_* > 1). The lower the value of R_L_, the higher the affinity of the adsorbent to the adsorbate. As revealed in Table 8, the R_L_ result attained in this study suggests the Langmuir isotherm model’s favorability.

(8) $$R_{L}=\frac{1}{1+(1+K_{L}+C_{o})}$$

The acquired maximum monolayer coverage capacity (*q_m_*) was 120.48 ${\mathrm{mg}\;g}^{-1}$ indicating the highest adsorption capacity of 1 g of Odaracha adsorbent.

#### 3.5.2. Freundlich Adsorption Isotherm Model for Pb(II)

**Figure 11:**
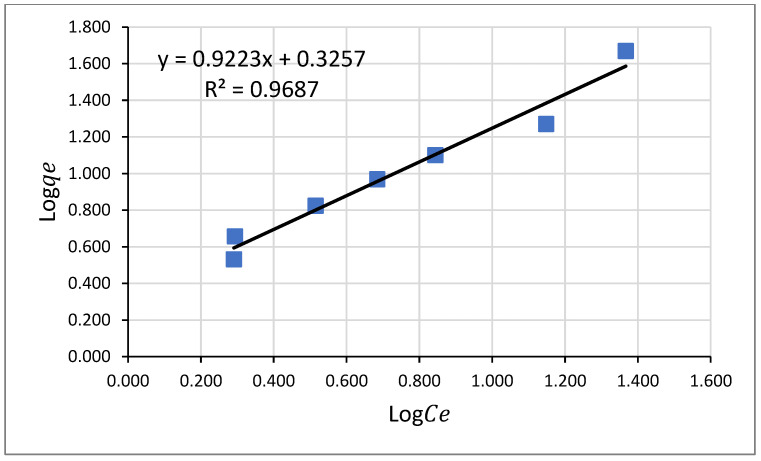
Freundlich Adsorption Isotherm Model for Lead.

The Freundlich adsorption model is based on equilibrium adsorption of adsorbate onto heterogeneous surfaces of the adsorbent, or it represents multilayer adsorption [53]. Its expression is given by Equation (9).

(9) $$logq_{e}=logK_{f}+\left(1/n\right)logC_{e}$$

where: *q_e_* is the amount of Pb^2+^ ions adsorbed at equilibrium per gram of the adsorbent (mg g^−1^), *K_f_* and n are the Freundlich adsorption model constants related to the adsorption capacity and intensity, respectively, and *Ce* is the final concentration of Pb^2+^ ion in equilibrium concentration (mg L^−1^). The adsorption capacity constant (*K_f_*) is calculated from the intercept, and the intensity constant (1/*n*) is figured from the slope of the linear plot shown in Figure 11.

If the value of n lies between 1 and 10, it designates favorable adsorption [54]. If 1/n is < 1, it designates normal adsorption, and if it is > 1, it implies cooperative adsorption [55]. As presented in Table 8, the value of 1/*n* obtained from this study was 0.9223, which indicates that the molecules responsible for the adsorption of Pb^2+^ onto Odaracha adsorbent favor adsorption, which indicates a chemisorption adsorption process. In another way, the value of the correlation coefficient result obtained from the plot validates the appropriateness of the Freundlich isotherm model to explain the adsorption process.

### 3.6. Comparison of Pb^2+^ Adsorption Capacity of Natural Adsorbents

Several scholars have investigated the adsorption capacity of different natural adsorbents, including clay minerals. The comparison between Odaracha adsorbent with various other adsorbents in terms of adsorption capacity for lead ions from an aqueous solution is presented in Table 9. In view of its uptake capacity, Odaracha can have a positive impact on the wastewater treatment systems.

## 4. Conclusions

In this study, the soil traditionally used by the local communities in the West Harerghe Zone of Oromia regional state was applied to remove lead contamination from wastewater. The available functional groups, the physical morphology of the soil, its physical and chemical properties, as well as the result of pHpzc identified the applicability of the adsorbent material for the adsorption of lead ions. The effect of experimental conditions such as contact time, pH, adsorbent dose, and initial concentration of adsorbate, on the removal efficiency of Pb^2+^ from synthetic wastewater was explored. In this regard, the study’s experimental results showed that the adsorption efficiency of Pb^2+^ by Odaracha adsorbent was positively associated with the contact time, solution pH, and adsorbent dosage, and negatively correlated with the initial concentration of the adsorbate. At optimum experimental conditions (i.e., 180 min contact time, pH 6, 15 g/L of adsorbent dose, and 30 mg/L of adsorbate concentration), Odaracha adsorbent removed 98.17% of lead ions from synthetic wastewater. The response surface method was implemented to develop a mathematical model to study the interaction effect of independent variables on Pb^2+^ removal efficiency of Odaracha adsorbent. The model’s adequacy and input parameter effects on the response variables were checked through analysis of variance evaluation of the P-value and F-value of the regression coefficient at a 95% confidence level. Pseudo-second-order adsorption kinetics were well shown with a high correlation coefficient of 0.998. A Freundlich adsorption isotherm was fitted with a 0.9687 correlation coefficient. In general, the study’s experimental result reveals the effectiveness and potential for Odaracha adsorbent in Pb^2+^ ion removal. Further, its effectiveness can be conceivably raised by extracting its active constituents at the industrial level.

## Figures and Tables

**Table 1:** Physicochemical Property of Odaracha Soil.

	Chemical Property								Physical Property		
	SiO_2_	Al_2_O_3_	Fe_2_O_3_	CaO	MgO	TiO_2_	H_2_O	LOI	Soil Texture %		
									Clay	Silt	Sand
Wt.%	30.94	8.71	5.18	25.12	1.44	0.3	<0.01	8.63	56.7	28.1	15.2

Source: Adapted from [27].

**Table 2:** Effect of contact time on Pb^2+^ removal efficiency (C_o_ = 70 mg/L, agitation speed = 150 rpm).

Contact Time (min)	pH	Dose (g/L)	C_f_ (mg/L)	Cr Adsorption %	q mg/g
60	6	15	5.905 ± 0.375	91.565	4.273
120	6	15	5.786 ± 0.217	91.735	4.281
180	6	15	1.964 ± 0.036	97.194	4.536
240	6	15	2.202 ± 0.206	96.854	4.520

**Table 3:** Effect of pH on Pb^2+^ removal efficiency (C_o_ = 70 mg/L, agitation speed = 150 rpm).

pH	Contact Time (min)	Dose (g/L)	C_f_ (mg/L)	Cr Adsorption %	q mg/g
3	120	15	7.202 ± 0.206	89.711	4.187
4	120	15	5.786 ± 0.217	91.735	4.281
5	120	15	5.774 ± 0.206	91.752	4.282
6	120	15	5.595 ± 0.055	92.007	4.294

**Table 4:** Effect of adsorbent dose on Pb^2+^ removal efficiency (C_o_ = 70 mg/L, agitation speed = 150 rpm).

Dose (g/L)	pH	Contact Time (min)	C_f_ (mg/L)	Cr Adsorption %	q mg/g
1	6	180	23.274 ± 0.206	66.752	46.726
5	6	180	6.976 ± 0.055	90.034	12.605
10	6	180	3.274 ± 0.412	95.323	6.673
15	6	180	1.964 ± 0.036	97.194	4.536

**Table 5:** Effect of initial concentration of adsorbate on Pb (II) removal efficiency of Odaracha adsorbent (agitation speed = 150 rpm).

Contact Time (min)	Dose (g/L)	pH	C_o_ (mg/L)	Av C_f_ (mg/L)	Pb Adsorption %	q mg/g
180	15	6	30	0.548 ± 0.021	98.175	1.963
180	15	6	50	1.238 ± 0.055	97.524	3.251
180	15	6	70	1.964 ± 0.036	97.194	4.536
180	15	6	90	3.071 ± 0.094	96.587	5.795
180	15	6	110	5.512 ± 0.135	94.989	6.966
180	15	6	130	13.869 ± 0.206	89.332	7.742
180	15	6	150	22.083 ± 0.149	85.278	8.528

**Table 6:** ANOVA results of the quadratic model for adsorption of Pb^2+^ by Odaracha.

Source	Sum of Squares	df	Mean Square	F-Value	*p*-Value	Remark
Model	2.966 × 10^−6^	9	3.295 × 10^−7^	32.71	<0.0001	Significant
A-Contact time	1.708 × 10^−6^	1	1.708 × 10^−6^	169.54	<0.0001	Significant
B-pH	2.667 × 10^−7^	1	2.667 × 10^−7^	26.48	<0.0001	Significant
C-Dose	2.925 × 10^−7^	1	2.925 × 10^−7^	29.03	<0.0001	Significant
AB	8.482 × 10^−8^	1	8.482 × 10^−8^	8.42	0.0099	Significant
AC	2.860 × 10^−8^	1	2.860 × 10^−8^	2.84	0.1103	Not significant
BC	4.000 × 10^−12^	1	4.000 × 10^−12^	0.0004	0.9843	Not significant
A^2^	2.588 × 10^−7^	1	2.588 × 10^−7^	25.69	<0.0001	Significant
B^2^	6.236 × 10^−8^	1	6.236 × 10^−8^	6.19	0.0235	Significant
C^2^	5.465 × 10^−9^	1	5.465 × 10^−9^	0.5425	0.4715	Not significant
Residual	1.713 × 10^−7^	17	1.007 × 10^−8^			
Lack of Fit	1.713 × 10^−7^	10	1.713 × 10^−8^	4.31	0.1402	Not significant
Pure Error	0.0000	7	0.0000			
Cor Total	3.137 × 10^−6^	26				

R^2^ = 0.9454, R^2^ _adjusted_ = 0.9165, R^2^ _predicted_ = 0.7866, AP = 20.1162, CV = 0.9143.

**Table 7:** Line fit model of Pseudo first and second-order kinetics for Pb^2+.^

Coagulant	Metal	First Order			
Odaracha	Lead	*q_e_* (exp) ${\mathrm{mg}\;g}^{-1}$	$q_{e}$ $(\mathrm{cal})\;{\mathrm{mg}\;g}^{-1}$	k1 $\left(\min^{-1}\right)$	R^2^
		4.536	2.482	$3.155\;\times \;10^{-2}$	0.5809
		**Second Order**			
		$q_{e}$ $(\exp)\;{\mathrm{mg}\;g}^{-1}$	$q_{e}$ $(\mathrm{cal})\;{\mathrm{mg}\;g}^{-1}$	k2 $\left({g\;\mathrm{mg}}^{-1}\min^{-1}\right)$	R^2^
		4.536	4.6339	$3.59\;\times \;10^{-2}$	0.9988

**Table 8:** Langmuir and Freundlich isotherm model constants for adsorption of Pb^2+.^

Adsorbent.	Metal	Langmuir			
Odaracha	Lead	$q_{m}\;{\mathrm{mg}\;g}^{-1}$	*K_L_*	$R_{L}$	R^2^
		120.48	0.0171	0.0139	0.9498
		**Freundlich**			
		$K_{f}$	1/n		R^2^
		2.117	0.9223		0.9687

**Table 9:** Comparison between Pb^2+^ adsorption capacities of various natural adsorbents and Odaracha soil.

Type of Adsorbents	Adsorption Capacity *q_m_* (mg/g)	References
Peat	9.489	[56]
Caladium bicolor (Wild Cocoyam)	88.5	[57]
PAC prepared from sugarcane bagasse	19.30	[58]
Bael Tree Leaf Powder	4.065	[32]
Montmorillonite	133	[59]
Natural bentonite	107	[60]
Natural Bentonite	32.67	[61]
Odaracha soil	120.48	This study

## Data Availability

The authors declare that the data supporting the findings of this study are available within the article, and other supplementary data are available from the corresponding author upon request.

## Sample Availability

Samples of the Odaracha soil are available from the authors.
